# The role of disease-linked residue glutamine-913 in support of the structure and function of the human electrogenic sodium/bicarbonate cotransporter NBCe1-A

**DOI:** 10.1038/s41598-018-20488-w

**Published:** 2018-02-15

**Authors:** Evan J. Myers, Aniko Marshall, Mark D. Parker

**Affiliations:** 1 Department of Physiology and Biophysics, Jacobs School of Medicine and Biomedical Sciences, University at Buffalo: The State University of New York, Buffalo, New York, USA; 2 Department of Ophthalmology, Jacobs School of Medicine and Biomedical Sciences, University at Buffalo: The State University of New York, Buffalo, New York, USA; 3 State University of New York Eye Institute, University at Buffalo: The State University of New York, Buffalo, New York, USA; 4 Present Address: University of Rochester Medical Center, School of Medicine and Dentistry, Rochester, New York, USA

## Abstract

Mutations in the sodium bicarbonate cotransporter NBCe1 (*SLC4A4*) cause proximal renal tubular acidosis (pRTA). We recently described a novel pRTA mutation p.Gln913Arg (Q913R), inherited in compound heterozygous form with p.Arg510His (R510H). Q913R causes intracellular retention of NBCe1 and a ‘gain of function’ Cl^−^ leak. To learn more about the importance of glutamine at position 913, we substituted a variety of alternative amino-acid residues (Cys, Glu, Lys, Leu, Ser) at position 913. Studying cRNA-injected *Xenopus* oocytes by voltage clamp, we find that most *de novo* mutants exhibit close-to-normal NBCe1 activity; only Q913K expresses a Cl^−^ leak. Studying transiently-transfected, polarised kidney cells by fluorescence microscopy we find that most *de novo* mutants (except Q913E) are intracellularly retained. A 3D homology model predicts that Gln913 is located in the gating domain of NBCe1 and neighbours the 3D space occupied by another pRTA-associated residue (Arg881), highlighting an important and conformationally-sensitive region of NBCe1. We conclude that the intracellular retention of Q913R is caused by the loss of Gln at position 913, but that the manifestation of the Cl^−^ leak is related to the introduction of Arg at position 913. Our findings will inform future studies to elucidate the nature and the consequences of the leak.

## Introduction

Blood plasma pH is maintained at a value close to 7.4. Deviations outside of this range (±0.05) are clinically defined as acidemia (↓pH) or alkalemia (↑pH). Bicarbonate (HCO_3_^−^) is an important physiological buffer. The ability of the CO_2_/HCO_3_^−^ buffer system to resist pH change hinges on the balance between the partial pressure of CO_2_ (*P*_CO2_) in the airspaces of the lungs and the concentration of HCO_3_^−^ in blood plasma.

$$HC{O}_{3}^{-}+{H}^{+}\leftrightarrow C{O}_{2}+{H}_{2}O$$

While respiration maintains a relatively constant arteriolar *P*_CO2_ (~40 mm.Hg), it is the action of the kidney that determines the concentration of bicarbonate in plasma (~24 mM). The majority of HCO_3_^−^ is generated in proximal tubule (PT) epithelial cells of each nephron and exported across the basolateral membrane of those cells, into circulation, via the electrogenic Na^+^/HCO_3_^−^ cotransporter NBCe1-A^1^. NBCe1-A in PT cells is also a major contributor to the reabsorption of ~80% of the HCO_3_^−^ filtered at the glomerulus. NBCe1-A is one of three major products of the *SLC4A4* gene; the others, NBCe1-B and NBCe1-C, are expressed in a variety of excitable and epithelial cells throughout the body and are primary engaged in HCO_3_^−^ import into cells. Control of intracellular [HCO_3_^−^], supports anion/fluid secretion and maintains neuronal excitability.

Recessively inherited mutations in NBCe1 cause proximal renal tubular acidosis (pRTA). pRTA is characterized by acidemia due to plasma HCO_3_^−^ insufficiency as well as a variety of disorders including developmental impairments, ocular abnormalities (band keratopathy, cataracts, and glaucoma), and neurological signs. Only fourteen such mutations have been described to date. These mutations (reported in the context of GenBank Accession NP_0037570) can be categorized as nonsense (p.Gln29X^2^ and p.Trp516X^3^), frame shift (p.Asn721ThrfsX30^4^ and p.Ser982AsnfsX4^5^), deletion (p.Leu738del.^6^) and missense (p.Arg298Ser^7,8^, p.Ser427Leu^9^, p.Thr485Ser^10^, p.Gly486Arg^11^, p.Arg510His^5,7,12^, p.Leu522Pro^13^, p.Ala799Val^10^, and p.Arg881Cys^5,10^). The fourteenth and most recently described mutation p.Gln913Arg is inherited in compound heterozygous form with p.Arg510His^14^. All mutations result in the loss of functional NBCe1 expression by decreasing NBCe1 abundance in the plasma membrane and/or Na^+^/2HCO_3_^−^ cotransport activity.

Two of these mutants exhibit a unique gain-of-function molecular phenotype: A799V exhibits a HCO_3_^−^-independent cation leak, while Q913R exhibits a Na^+^ and HCO_3_^−^-independent Cl^−^ leak. A homology model of NBCe1, based on the crystal structure of the related protein AE1 (anion exchanger 1, encoded by *SLC4A1*), reveals that Ala799 is located in the substrate coordinating region in the core of the ion translocating domain^15^. We now extend our original study to investigate the potential functional importance of Gln913. In the present report, we study the electrophysiological and trafficking characteristics of a series of *de novo* NBCe1 substitution mutants (Q913C, Q913E, Q913K, Q913L, and Q913S chosen to represent a diversity of chemical and structural characteristics, see Methods) in order to determine the extent to which the molecular defects associated with Q913R result from the loss of Gln or from the introduction of Arg at position 913. Also for the first time, we discuss the location of Gln913 in a 3D homology model of NBCe1 and its implications for our findings.

## Results

### Q913X mutants are capable of Na^+^/2HCO_3_^−^ cotransport

**Figure 1:**
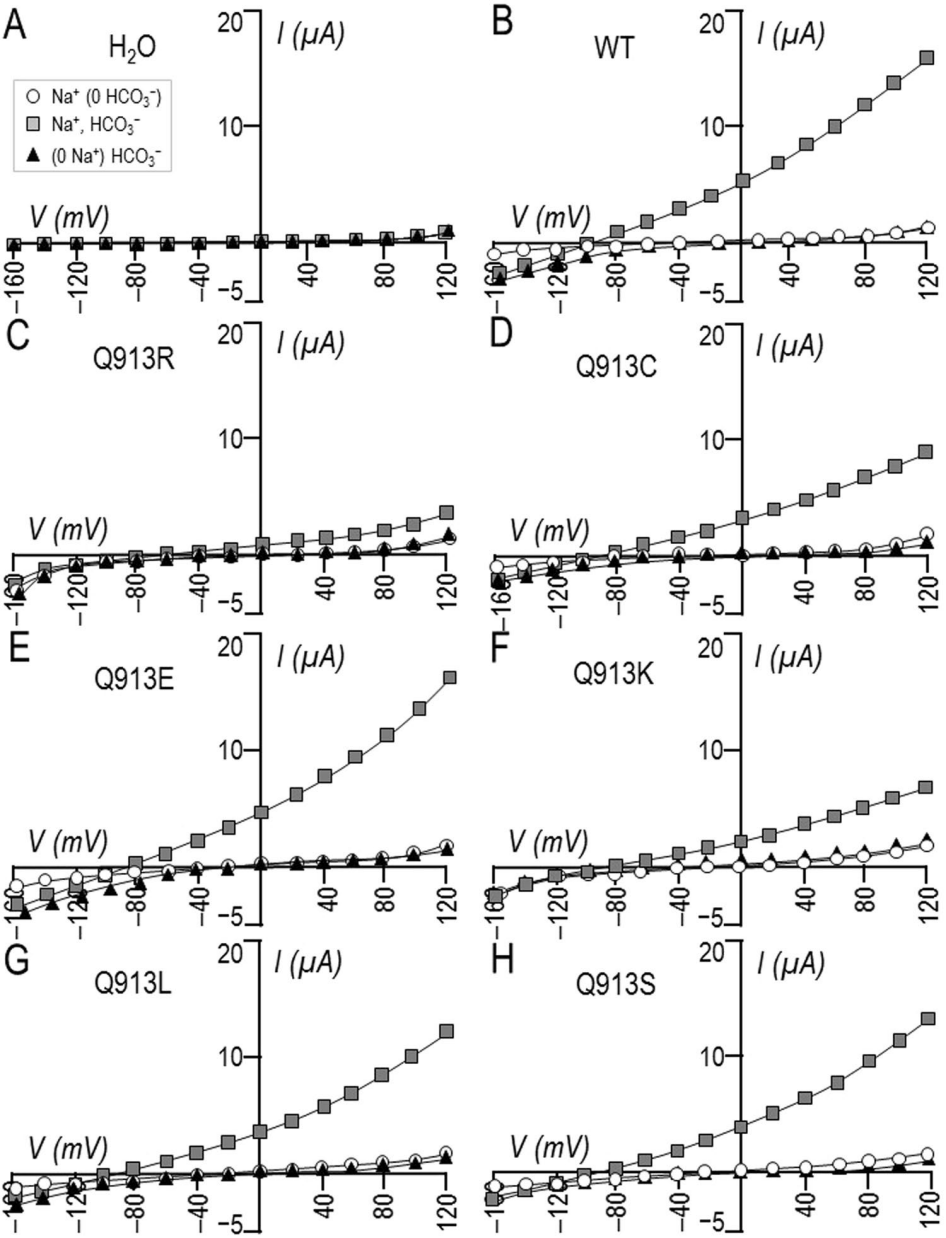
Effect of Na^+^ and HCO_3_^−^ replacement on currents mediated by WT or Q913X mutants in *Xenopus* oocytes. (**A**) Representative current-voltage (I–V) relationship from a H_2_O-injected oocyte as it was sequentially exposed to ‘Na (0 HCO_3_)’, ‘Na, HCO_3_’, and ‘(0 Na) HCO_3_’ solutions. (**B**–**H**) Equivalent data from oocytes expressing WT, Q913R, Q913C, Q913E, Q913K, Q913L, or Q913S.

**Figure 2:**
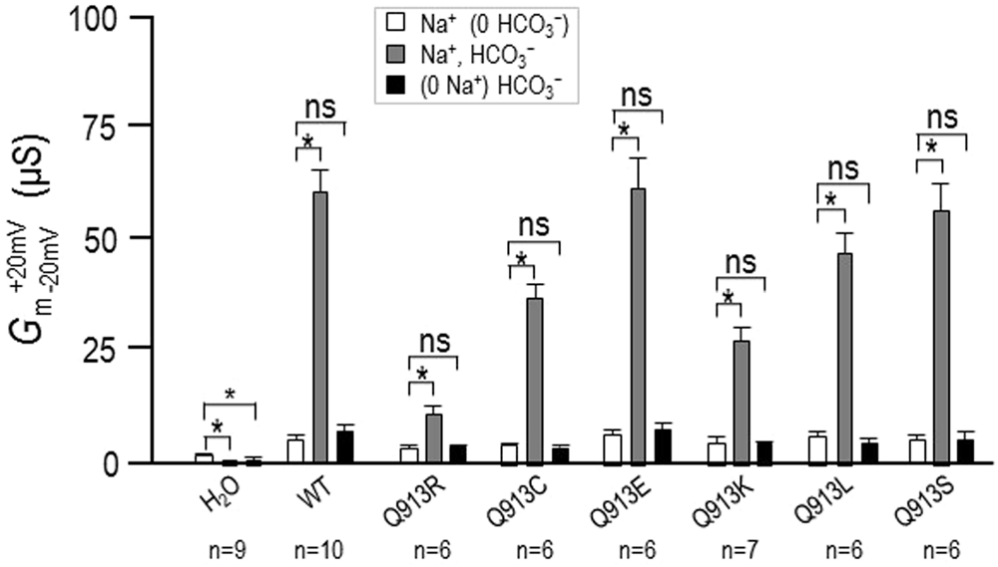
Membrane conductance (*G*_m_) of oocytes expressing WT or Q913X mutants ±NaHCO_3_. Bar chart shows the average *G*_m_ values, measured between −20 mV and +20 mV, calculated from a larger set of *I*-*V* relationships such as those shown in Fig. 1. ‘*’denotes statistical significance between bars according to a paired one-tailed t-test (P < 0.006, accounting for Bonferroni correction for eight comparisons). ‘ns’ demotes no significance between bars by the same analysis.

Figure 1A shows three *I*-*V* relationships gathered from a H_2_O-injected oocyte as it was sequentially superfused with ‘Na (0 HCO_3_)’, ‘Na, HCO_3_‘, and ‘(0 Na) HCO_3_’ solutions. The conductance of the plasma membrane (*G*_m_) described by the slope of these lines was not greatly affected by the solution changes, although the small changes that we did observe are statistically significant as shown by the data from a greater number of H_2_O-injected oocytes in Fig. 2. Figures 1B and 2 show equivalent data from oocytes expressing wild-type NBCe1-A-EGFP (WT). In contrast to the behaviour of H_2_O-injected cells, the presence of HCO_3_^−^ in the superfusate caused a large and significant increase in *G*_m_ for WT-expressing cells (white vs gray symbols) that was cancelled upon removal of Na^+^ from the perfusate (black symbols): a pattern consistent with electrogenic Na^+^- and HCO_3_^−^-dependent transport. Figure 1C–H show equivalent data from single oocytes expressing either the pRTA-associated mutant Q913R or any one of the *de novo* mutants Q913C, E, K, L, or S. All mutants exhibited the signature pattern of electrogenic Na^+^- and HCO_3_^−^-dependent transport with varying levels of robustness (Fig. 2). We will consider the relative magnitude of these conductances among groups of oocytes in a later section.

**Figure 3:**
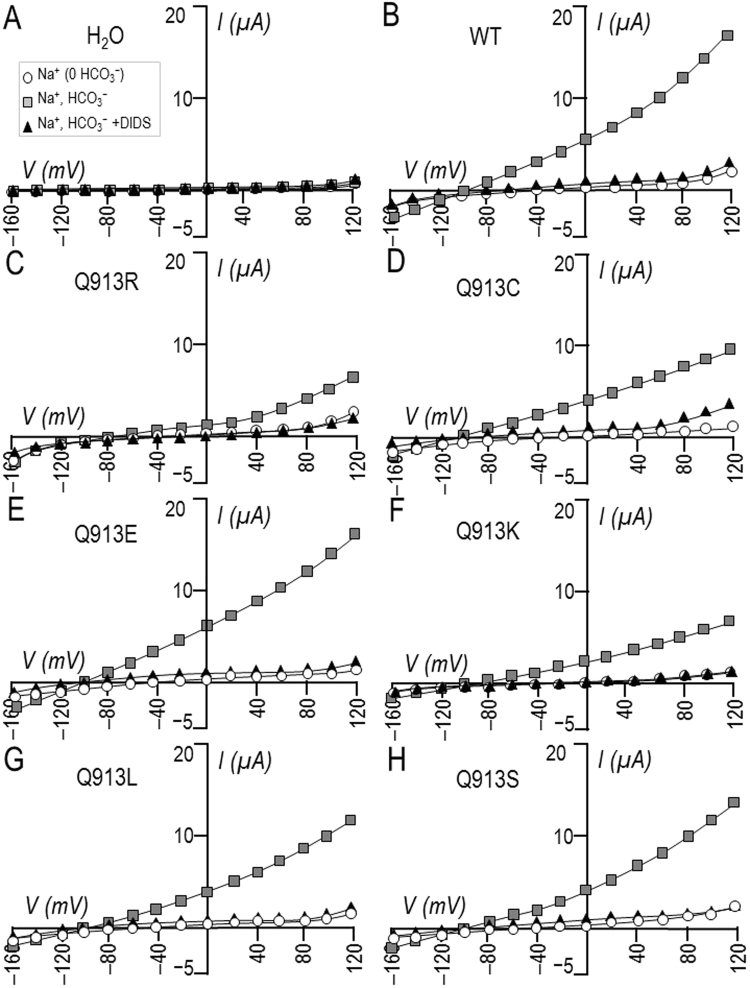
Effect of DIDS on currents mediated by WT or Q913X mutants in *Xenopus* oocytes. (**A**) Representative current-voltage (I–V) relationship from a H_2_O-injected oocyte as it was sequentially exposed to ‘Na (0 HCO_3_)’, ‘Na, HCO_3_’, and ‘Na, HCO3, +200 µM DIDS’ solutions. (**B**–**H**) Equivalent data from oocytes expressing WT, Q913R, Q913C, Q913E, Q913K, Q913L, or Q913S.

**Figure 4:**
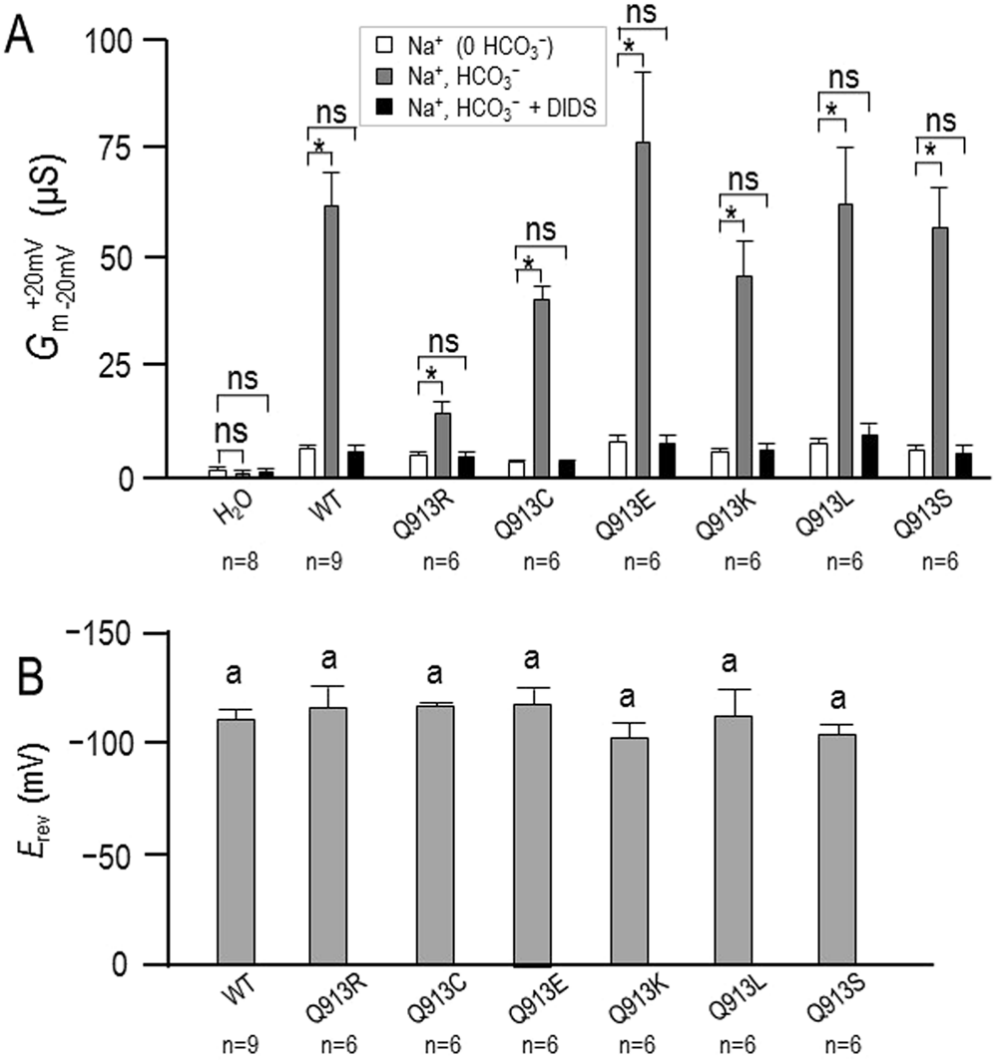
Membrane conductance (*G*_m_) of oocytes expressing WT or Q913X mutants ± DIDS. (**A**) Bar chart shows the average *G*_m_ values, measured between −20 mV and + 20 mV, calculated from a larger set of *I*-*V* relationships such as those shown in Fig. 3. ‘*’ denotes statistical significance between bars according to a paired one-tailed t-test (P < 0.006, accounting for Bonferroni correction for eight comparisons). ‘ns’ demotes no significance between bars by the same analysis. (**B**) Reversal potential of the DIDS-sensitive transport process as reported by the intersection of the I-V plots ± DIDS in data such as that in Fig. 3. ‘a’ denotes that all groups were statistically indistinguishable by ANOVA with post hoc Tukey analysis, 95% confidence limit.

Figure 3A–H show, for each group of oocytes, three *I-V* relationships gathered from single oocyte as they were sequentially superfused with ‘Na (0 HCO_3_)’, ‘Na, HCO_3_’, and ‘Na, HCO_3_+ DIDS’ (DIDS: 4,4’-Diisothiocyano-2,2’-stilbenedisulfonic acid) solutions. 1 min exposure to 200-µM DIDS cancelled the HCO_3_^−^-dependent increase in *G*_m_ for each NBCe1-expressing cell, as shown in the averaged data presented in Fig. 4A. The intersection of the *I*-*V* relationships gathered in ‘Na, HCO_3_’ solution ± DIDS reports the reversal potential (*E*_rev_) of the transport process, which is a proxy for the Na^+^:HCO_3_^−^ cotransport stoichiometry^16^. Figure 4B shows that *E*_rev_ is statistically indistinguishable among the *de novo* mutants, WT, and Q913R. The calculated value of *E*_rev_ is consistent with a 1 Na^+^:2 HCO_3_^−^ cotransport stoichiometry^16,17^.

### Q913X mutants exhibit close-to-normal per-molecule Na^+^/2HCO_3_^−^ cotransport activity

**Figure 5:**
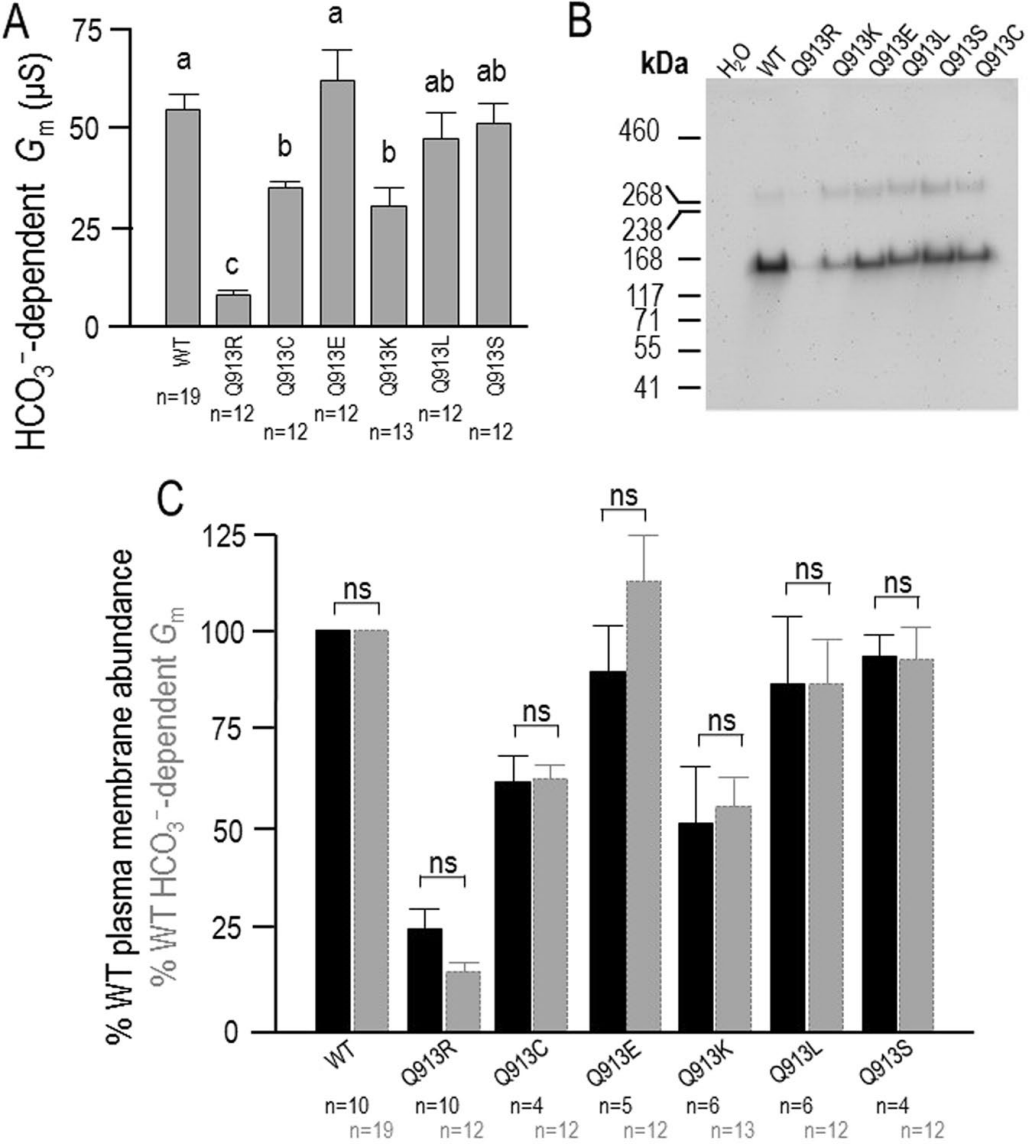
Functional expression of WT or Q913X mutants in *Xenopus* oocytes. (**A**) HCO_3_^−^-dependent *G*_m_ defined as *G*_m_ in ‘Na, HCO_3_’ solution less the *G*_m_ in ‘Na (0 HCO_3_)’ solution, pooled from Figs 2 and 4A. Groups that do not share the same annotated letter are deemed significantly different by ANOVA with post hoc Tukey analysis, 95% confident limit. (**B**) A representative anti-EGFP western blot of isolated membrane (biotinylated) fractions from *Xenopus* oocytes injected with H_2_O, or cRNA encoding WT or a Q913X mutant. (**C**) Bar chart of average EGFP intensity signal from western blot of biotinylated fractions of Q913X-expressing oocytes (black bars), such as that in Fig. 5B, normalized to the signal from WT-expressing oocytes from each blot. Data are plotted alongside a similarly normalized version of the data presented in Fig. 5A (gray bars). ‘ns’ denotes no significance between bars (P > 0.007, accounting for Bonferroni correction for seven comparisons).

Figure 5A shows the combined HCO_3_^−^-dependent *G*_m_ values (i.e., *G*_m_ in ‘Na,HCO_3_’ solution less the *G*_m_ in ‘Na (0 HCO_3_)’ solution) pooled from the data in Figs 2 and 4A. ANOVA analysis reports that (1) cells expressing Q913E, Q913L, and Q913S exhibit a HCO_3_^−^-dependent *G*_m_ that is indistinguishable from that of cells expressing WT, (2) cells expressing Q913C, Q913K, and Q913R exhibit a HCO_3_^−^ dependent *G*_m_ that is smaller than that of cells expressing WT, and (3) cells expressing the pRTA-associated mutant Q913R exhibit the smallest HCO_3_^−^ dependent *G*_m_. In order to relate these values to a ‘per-molecule’ activity, we need to determine the relative abundance of each transporter clone in the oocyte plasma membrane. Figure 5B shows a representative western blot of biotinylated (i.e., plasma-membrane resident) NBCe1-A protein from H_2_O-injected oocytes as well as from oocytes expressing WT, Q913R, Q913C, Q913E, Q913K, Q913L, and Q913S. In all cells expressing NBCe1-A constructs, the protein migrated as two bands: a major band of ~ 168 kDa representing monomeric NBCe1-A-EGFP, and a minor band greater than 268 kDa consistent with the expected molecular weight of dimeric NBCe1-A-EGFP^17^. From a larger number of biological replicate samples probed on similar blots, we calculated the relative abundance of each mutant (dimer plus monomer) in the plasma membrane versus WT (Fig. 5C, black bars). Reflecting the *G*_m_ data in Fig. 5A, Q913R exhibits the weakest expression. The gray bars in Fig. 5C reproduce the *G*_m_ data from Fig. 5A, normalized to the average *G*_m_ of WT-expressing cells. Statistical analysis (unpaired, two-tailed t-test with Bonferroni correction for multiple comparisons) reports no significant disparity between the relative plasma-membrane expression (black bars) and the relative HCO_3_^−^-dependent *G*_m_ (gray bars) for any of the mutants.

### Q913K, like Q913R, exhibits an additional anion leak

**Figure 6:**
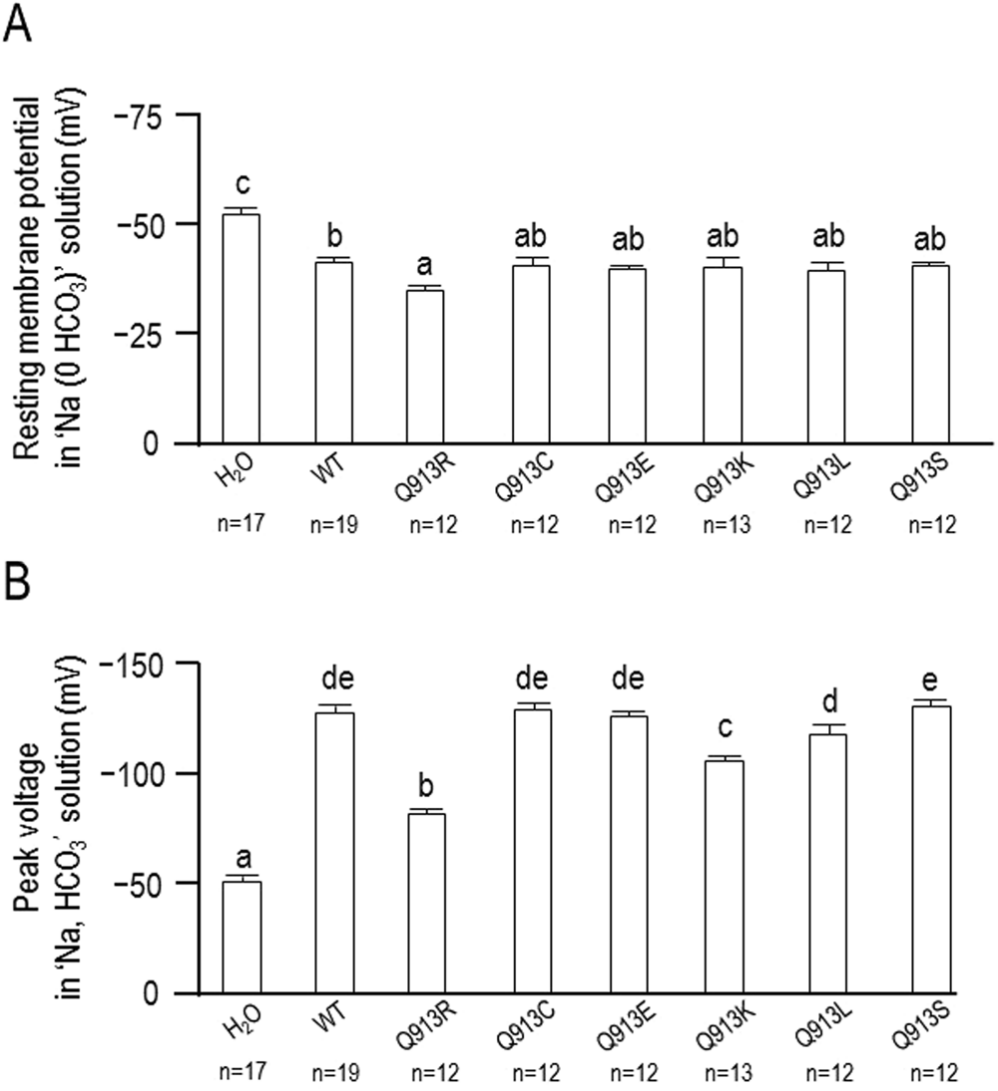
Membrane potential (*V*_m_) of oocytes expressing WT or Q913X mutants. (**A**) Average *V*_m_ of cells bathed in ‘Na (0 HCO_3_)’ solution. (**B**) Average of the most negative values of *V*_m_ achieved upon exposure of cells to ‘Na, HCO_3_’ solution. Within each panel, groups that do not share the same annotated letter are deemed significantly different by ANOVA with post hoc Tukey analysis, 95% confident limit.

Prior to each of the experiments in Figs 1–4 we measured the spontaneous membrane potential (*V*_m_) of the cells at rest while bathed in ‘Na (0 HCO_3_)’ solution (Fig. 6A).We found that the average *V*_m_ for cells expressing any of the NBCe1 constructs was significantly more depolarized than the *V*_m_ of H_2_O-injected oocytes (Fig. 6A). Among the cells expressing a mutant NBCe1 construct, only those expressing Q913R exhibited a *V*_m_ that was statistically distinguishable (more depolarized) from the *V*_m_ of cells expressing WT (Fig. 6A).

The application of ‘Na, HCO_3_’ solution did not affect the *V*_m_ of H_2_O-injected cells (P = 0.68, paired, two-tailed t-test, not shown). The same maneuver caused a significant and substantial hyperpolarization of average *V*_m_ for all groups of cells expressing an NBCe1 construct (paired, one-tailed t-test with Bonferroni correction, not shown) as *V*_m_ tends towards *E*_rev_ for NBCe1. The maximum extent of hyperpolarization (i.e., most negative *V*_m_) observed upon exposure to ‘Na, HCO_3_’ solution is shown in Fig. 6B. ANOVA reports that cells expressing Q913C, Q913E, Q913L, and Q913S hyperpolarized to the same extent as cells expressing WT. However, cells expressing Q913R or Q913K did not hyperpolarize to the same extent as cells expressing WT.

**Figure 7:**
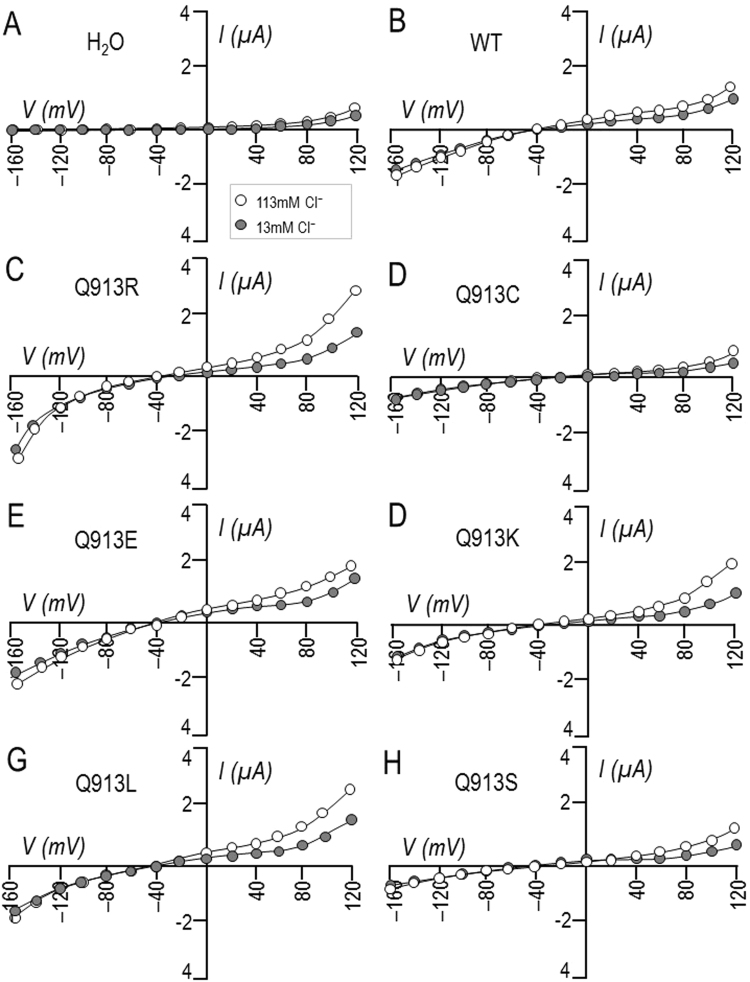
Effect of Cl^−^ replacement on currents mediated by WT or Q913X mutants in *Xenopus* oocytes in the absence of HCO_3_^−^. (**A**) Representative current-voltage (I–V) relationship from a H_2_O-injected oocyte as it was sequentially exposed to ‘Na (0 HCO_3_)’ solution containing either 113 mM Cl^−^ or 13 mM Cl^−^. (**B**–**H**) Equivalent data from oocytes expressing WT, Q913R, Q913C, Q913E, Q913K, Q913L, or Q913S.

**Figure 8:**
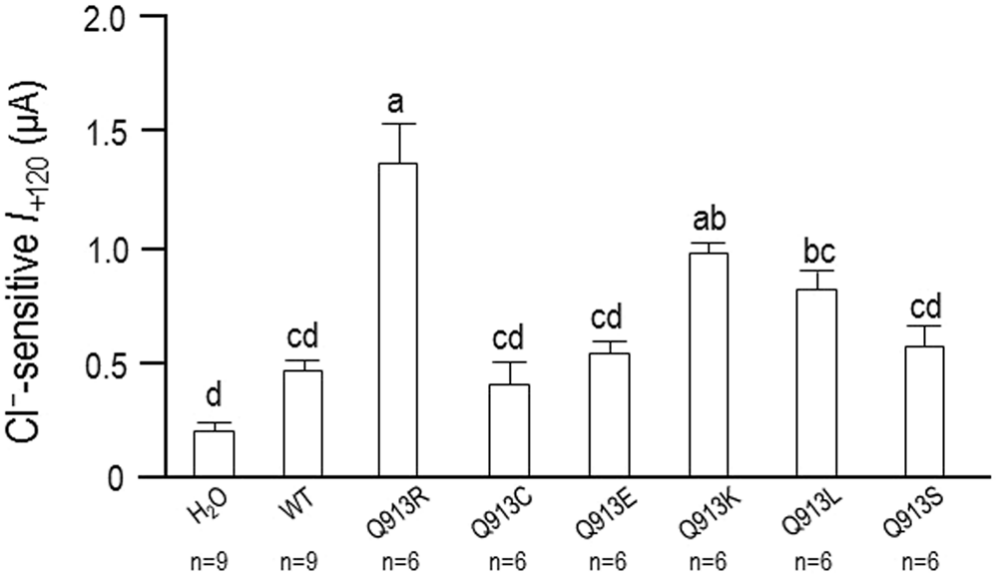
Cl^−^-sensitivity of the membrane current (*I*) of oocytes expressing WT or Q913X mutants. Bar chart shows the average decrease in current, measured at +120 mV, upon lowering of extracellular Cl^−^ from 113 mM to 13 mM calculated from a larger set of *I*-*V* relationships such as those shown in Fig. 7. Groups that do not share the same annotated letter are deemed significantly different by ANOVA with post hoc Tukey analysis, 95% confident limit.

We have previously shown that the hyperpolarization deficit exhibited by Q913R is reflective of the presence of a HCO_3_^−^-independent anion leak that prevents electrogenic NBCe1 activity from dominating *V*_m_. To determine whether any of the de novo mutants exhibit this unusual anion leak, we examined the effect of lowering extracellular [Cl^−^] on the HCO_3_^−^-independent currents associated with NBCe1 expression. Figure 7A–H shows representative *I*-*V* relationships obtained from single oocytes during a set of experiments in which oocytes were superfused with ‘Na (0 HCO_3_)’ solution (containing 113 mM Cl^−^) followed by a reduced-Cl version of the same solution that contains only 13 mM Cl^−^. This maneuver is anticipated to reduce the magnitude of the outward currents (i.e., those above the x-axis) that represent Cl^−^ influx. Figure 8 shows the average reduction in current at +120 mV (*I*_+120_) from a greater number of these experiments. ANOVA discloses that (1) cells expressing Q913R, Q913K, or Q913L exhibit Cl-sensitive currents that are significantly greater than those endogenously expressed by H_2_O-injected oocytes and (2) only cells expressing Q913R or Q913K exhibit Cl-sensitive currents that are significantly greater than those exhibited by cells expressing WT.

### Q913X mutants—except Q913E—are withheld from the basolateral membrane of polarised renal epithelia

**Figure 9:**
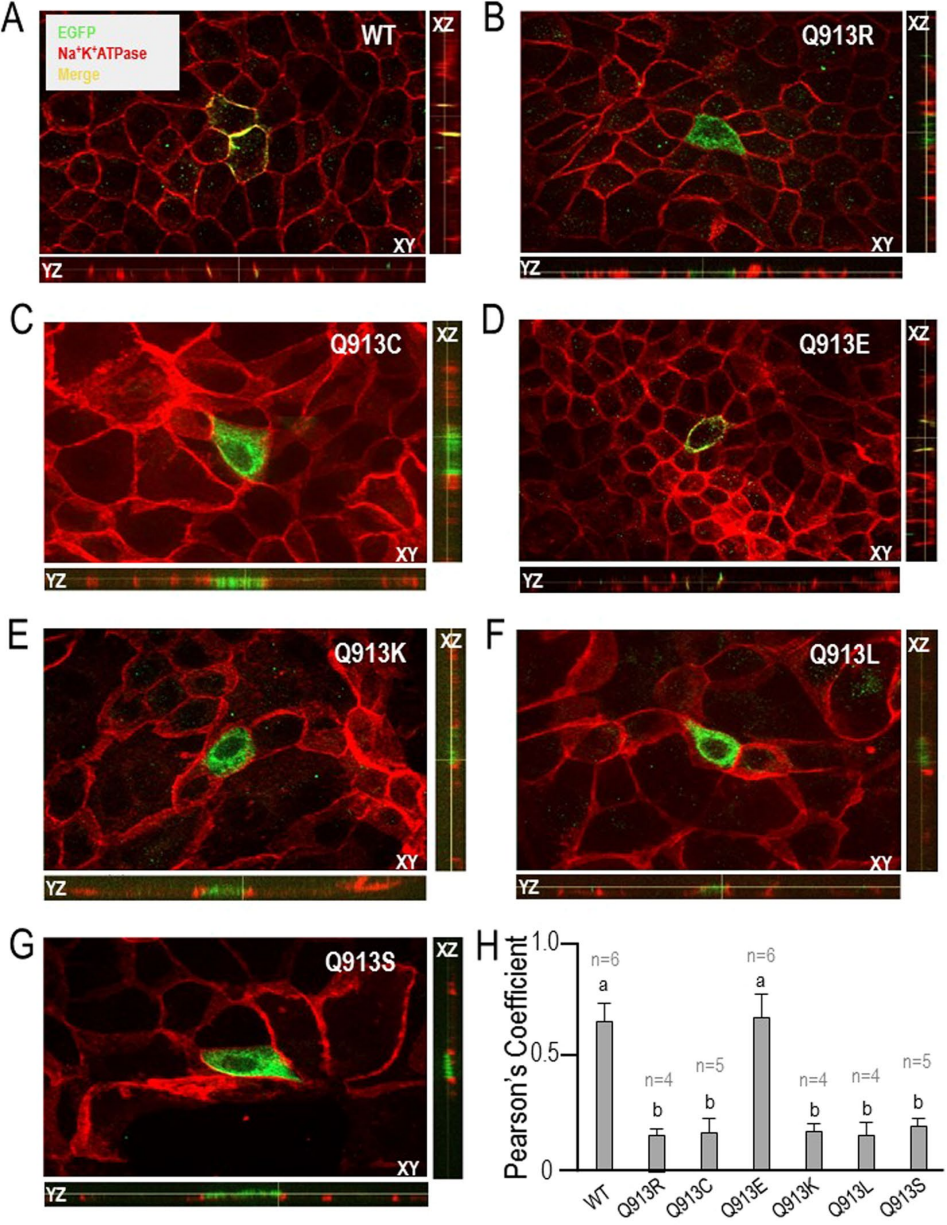
Distribution of WT or Q913X mutant NBCe1-A-EGFP in polarised MDCK−II Cells. (**A**) Representative WT transfected cells showing the distribution of NBCe1-EGFP and Na^+^-K^+^ ATPase in the XY, XZ, and YZ plane as disclosed by an anti-EGFP primary antibody following by an Alexa-488 conjugated secondary antibody (green) and anti-Na^+^-K^+^ ATPase followed by an Alexa-594 conjugated secondary antibody (red). (**B**–**G**) Equivalent representative from cells transfected with Q913R, Q913C, Q913E, Q913K, Q913L, and Q913S. (**H**) Bar chart showing the average Pearson’s coefficient values for co-incidence of EGFP immunoreactivity with Na^+^-K^+^ ATPase immunoreactivity. Groups that do not share the same annotated letter are deemed significantly different by ANOVA with post hoc Tukey analysis, 95% confident limit.

Figure 9A–G shows example fluorescence micrographs and z-axis projections of polarised MDCK-II cells (MDCK: Madin Derby Canine Kidney cell line) that were transiently transfected either with WT, Q913R, or one of the *de novo* Q913X mutants. Figure 9A shows that WT (disclosed by EGFP immunoreactivity, green) exhibits a peripheral distribution that substantially overlaps (yellow) with that of the basolateral marker Na^+^/K^+^-ATPase (red), consistent with lateral expression of WT. Figure 9B shows that the distribution of Q913R does not substantially overlap with the location of Na^+^/K^+^-ATPase, consistent with the previous report of enhanced intracellular retention for Q913R^14^. Of the *de novo* mutants (Fig. 9C–G) Q913C, Q913K, Q913L, and Q913S exhibit a distribution pattern that is similar to that exhibited by Q913R, while Q913E exhibits an expression pattern that is similar to that exhibited by WT (Fig. 9D). Figure 9H shows a quantitation of the co-incidence of EGFP and Na^+^/K^+^-ATPase immunoreactivity from a larger number of cells in each group, for which a Pearson’s coefficient of 1.0 denotes perfect co-incidence of EGFP immunofluorescence with immunofluorescence of our basolateral marker (Na^+^,K^+^-ATPase). The analysis shows that the distribution of Q913C, Q913K, Q913L, and Q913S is not significantly different from that of Q913R (i.e., predominantly intracellular) and that the distribution of Q913E is not significantly different from that of WT.

### Gln913 is located in the gating domain of NBCe1

**Figure 10:**
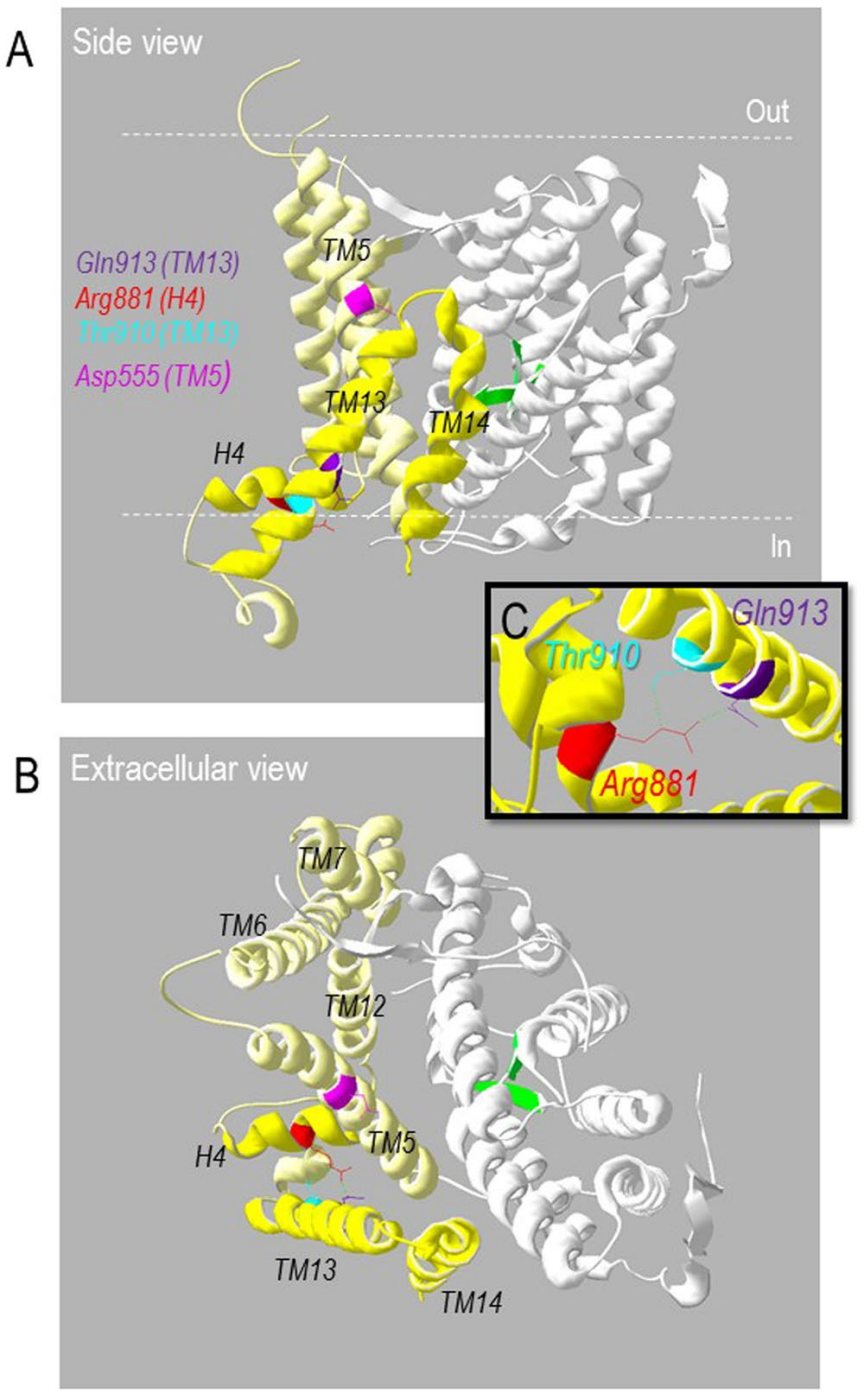
Homology model of the transmembrane domain of NBCe1. (**A**) Side view, of an NBCe1 homology model, based on the crystal structure of AE1 (PDB ID: 4YZF) showing the relative positions of the gating domain (yellow; H4, TM13, and TM14 are colored darker yellow to provide contrast), anion translocating core domain (white), putative substrate interacting domain (green), Gln913 (purple), Arg811 (red), Thr910 (cyan), and Asp555 (magenta). The gating domain is composed of six transmembrane spans (TM5-7, TM12-14) and a hydrophilic helix (H4) in the structured intracellular loop between TM12 and TM13. (**B**) View of the intracellular face of the same model. (**C**) Closer view of putative hydrogen bonds (green dotted-lines) between Arg881, Thr910, and Gln913.

In order to place our observations into a structural context, we generated a homology model of NBCe1 based upon the recently published 3.5-Å AE1 crystal structure^18^. Figure 10A and B show two elevations of NBCe1 colored to show the putative locations of the gating domain (yellow), the substrate-translocating core domain (white) and the substrate-interacting region (green). Gln913 (purple) is situated at the cytoplasmic end of transmembrane span 13 (TM13), in the periphery of the gating domain. The side chain of Gln913 is predicted to have no direct interaction with the substrate-translocating core domain or the dimer interface (which is at the left-hand side of Fig. 10). The Gln913 side-chain group faces the cytoplasmic end of TM5 as well as hydrophilic helix 4 (H4) in the structured loop between TMs 12 and 13: a similar space to that occupied by Arg881 (red). Arg881 is predicted to form a hydrogen bond with Thr910 (cyan), as their equivalents do in the AE1 structure. Although the AE1-equivalents of Gln913 and Arg881 do not form a hydrogen bond in the AE1 crystal structure, some Gln913 rotamers can hydrogen bond with Arg881 in the NBCe1 homology model (e.g., Fig. 10C: the length of the bond between Arg881 and Gln913 is 2.9 Å which is sufficiently close to constitute an energetically significant interaction^19^). When the substitutions considered in the present study are introduced into the model, only p.Q913E conserves the H-bond without introducing a steric clash (not shown). Also notable in Fig. 10 is the location of Asp555 in TM5 (pink): the *de novo* mutant D555E is also leaky to Cl^− ^^20^.

## Discussion

Gln913 of NBCe1-A is mutated in pRTA so we set out to investigate the importance of Gln913 by investigating the amino-acid substitution tolerance at this position. Note that to distinguish discussion of the mutants (mutant proteins) and the mutations (mutated amino acids) the discussion, we refer to the mutants as ‘Q913C’, ‘Q913E’, ‘Q913K’, ‘Q913L’, and ‘Q913S’ and we will refer to the mutations as ‘p.Q913C’, ‘p.Q913E’, ‘p.Q913K’, ‘p.Q913L’, and ‘p.Q913S’.

### The importance of Gln913 for electrogenic Na^+^/2HCO_3_^−^ cotransport

Like WT and Q913R, all five of the *de novo* mutants that we generated for this study are capable of mediating a Na^+^ and HCO_3_^−^ dependent conductance that is blocked by 200 µM DIDS. Furthermore, *E*_rev_ is not altered by the *de novo* substitutions indicating that the Na^+^/2HCO_3_^−^ stoichiometry is unchanged by these mutations. Although the magnitude of the HCO_3_^−^-dependent conductances varied among mutants, we found that these variations closely reflected differences in plasma-membrane expression among the mutants. These data are consistent with the hypothesis that the per-molecule Na^+^/2HCO_3_^−^ cotransport activity is unaffected by these substitutions, as we had previously described for Q913R. Because such a variety of substitutions (nonpolar-aliphatic, polar-uncharged, positively-charged, and negatively-charged R-groups) are tolerated at this position, we conclude that Gln913 plays no discernable role in support of the electrogenic Na^+^/2HCO_3_^−^ cotransport action of NBCe1.

### The importance of Gln913 for basolateral presentation of NBCe1-A

We have previously shown that the Q913R (as well as the R510H) mutant is retained from the plasma membrane in polarised MDCK-II cells and concluded that NBCe1 mis-targeting is the underlying cause of pRTA in the individual with the compound heterozygous mutations p.Q913R/p.R510H. However we did not determine whether (1) it is the presence of Gln913 that is required for normal trafficking of NBCe1-A or (2) it is the aberrant presence of Arg913 that causes the defect. In the present study we find that four of the five *de novo* substitutions (p.Q913C, p.Q913K, p.Q913L, p.Q913S) result in enhanced intracellular retention of NBCe1-A: only Q913E appears to accumulate normally in the basolateral membrane. It is likely that p.Q913E is well tolerated because Gln and Glu are very similar in size and differ only in the substitution of an amine for a hydroxyl group. Because less conservative substitutions are poorly tolerated at this position, we conclude that Gln913 (or at least the features that it shares with Glu) is required for the plasma membrane accumulation of NBCe1. It is unclear whether non-conservative substitutions at 913 disrupt a specific localization signal, although we note that none has been described at this location for SLC4 members. It seems more likely that mutations cause a localized misfolding that is sufficient to be recognized by the cell’s protein sorting quality control machinery but that is not sufficient to interfere with NBCe1 transport action.

On a technical note, a failure of NBCe1 mutants to accumulate in the oocyte plasma membrane typically presages intracellular retention of those mutants in polarised MDCK cells, as has been previously described for R510H, A799I, A799V, R881C, and Q913R^14,17,21^ and is presently described for Q913C and Q913K. However in the present study Q913L and Q913S are intracellularly retained in MDCK cells yet accumulate normally in the oocyte plasma membrane. Thus, contrary to previous indications, plasma membrane accumulation in oocytes is not a reliable indicator of the behaviours of that protein in polarised mammalian cells.

### The influence of the residue at position 913 on expression of a Cl^−^ leak

Beyond intracellular retention, a second unusual feature of Q913R is a HCO_3_^−^-independent Cl^−^ leak. As previously described, this leak manifests in several ways: (1) In the absence of HCO_3_^−^, Q913R-expressing cells are more depolarized than WT-expressing cells, (2) In the presence of HCO_3_^−^, the *V*_m_ of Q913R-expressing cells is not dominated by Na^+^/2HCO_3_^−^ cotransport activity, despite expression of adequate HCO_3_^−^-dependent conductance, and (3) Q913R-expressing cells exhibit a greater Cl^−^-dependent current than WT-expressing cells. In the present, only cells expressing Q913R exhibited the first trait and only cells expressing Q913R or Q913K exhibited the latter two traits. When we take into account the magnitude of the Cl^−^-dependent currents and the plasma-membrane abundance of the mutants in oocytes, it is clear that the per-molecule Cl^−^-leak through Q913K is less than 50% of that through Q913R, which may contribute towards the lack of influence of Q913K upon *V*_m_ in HCO_3_^−^-free solution. Although Q913C, Q913L, and Q913S show no significant indications of being leaky, we note that cells expressing Q913L did on average display a tendency to hyperpolarize less than WT-expressing cells in HCO_3_^−^-solution and to exhibit larger Cl^−^-dependent currents than WT-expressing cells. Again, taking the plasma-membrane abundance of Q913L vs Q913R into account, it is likely that if Q913L did express a leak, it would be an order of magnitude smaller than that exhibited by Q913R and consequently difficult to reliably detect. These data indicate that it is the specific introduction of a bulky and/or positively-charged residue at 913 that causes the exhibition of the leak, although we cannot determine which of those two factors are critical due to the limitations of naturally-occurring amino-acid residues with which to substitute. Thus is seems unlikely that the leak pathway is a cryptic feature of WT that could be unveiled in response to pathophysiological stimuli such as cell swelling, as has been described for a Cl^−^ leak that is native to trout AE1^22,23^.

### Structural predictions regarding position 913

The peripheral location of Gln913 in the gating domain of NBCe1, away from the putative determinants of substrate translocation and the gating/core domain interface, is consistent with the lack of influence of Gln913 substitution on substrate translocation. The Gln913 side-chain is buried within the structure making its replacement likely to affect local structure; with larger substituents presumed to be most disruptive. Only Glu can be substituted in its place without defect, or without disrupting the putative hydrogen bond with Arg881, suggesting the size and carboxyl group of Gln913 are more important than its amine group (which is replaced by a hydroxyl group in Glu). The similar location of, and perhaps even interaction between, Arg881 and Gln913 suggests that a common structural derangement underlies the presently described mistargeting of Q913X mutants and the previously described mistargeting of R881C in mammalian cells^21^. Like Q913X, R881C also exhibits apparently normal per-molecule Na^+^/2HCO_3_^−^ cotransport activity^21^. The model does not permit us to determine a pathway for the Cl^−^ leak associated with Q913R but we can consider three possibilities (1) local misfolding (such as might occur due to steric clashes or charge repulsions between Arg913 and neighboring residues such as Arg881) transfers to the ion translocation core domain allowing Cl^−^ to slip through the substrate translocation gate. This seems least likely as ion translocation is not detectably altered. (2) Local misfolding allows Cl^−^ to leak between the core and gating domains, similar to the pathological gating pore currents that arise in voltage-gated Na^+^ channels mutated in periodic paralysis^24^. (3) Local misfolding opens a conduit at the monomer-monomer interface within the NBCe1 dimer. Interestingly, a similar voltage-dependent anion leak is exhibited by a conservative *de novo* mutation p.D555E in TM5 of NBCe1-A^20^. It is intriguing to speculate that the introduction of Arg/Lys at position-913, into a tightly-constrained space that faces TM5, causes a relocation of D555, resulting in the opening of the same or similar anion conduit that is a feature of D555E.

## Conclusion

Gln913 is a critical structural component of NBCe1 that is required for the optimal folding of NBCe1, but Gln913 is not required for the Na^+^/2HCO_3_^−^ cotransport action of NBCe1. Most substitutions at this site would be expected to cause pRTA by virtue of intracellular retention of NBCe1, but manifestation of the Cl^−^ leak is specific to the pRTA mutant Q913R and the similar *de novo* mutant Q913K. The variety of Q913X molecular phenotypes identified in this study and the identified commonalities between Gln913, Arg881, Asp555 and their mutants will allow a better understanding of the nature, path, and consequences of the anion-leak pathway in future transgenic animal and molecular dynamic simulation studies.

## Methods

### cDNA Clones

The construction of the plasmids for expression of wild-type NBCe1-A with a carboxy-terminal enhanced green fluorescent protein (EGFP) tag in mammalian cells (NBCe1-A-EGFP.pcDNA3.1) and EGFP-tagged NBCe1-A in *Xenopus* oocytes (NBCe1-A-EGFP.pGH19), and the introduction to each of the Q913R mutation, has been described previously^14^. Other codon substitutions at position 913 (underlined below, collectively referred to as Q913X, where X is any amino acid:)^25^ were introduced using a QuikChange site-directed mutagenesis kit (Agilent Technologies Inc., Santa Clara, CA) using the following primers with their reverse complements: 5′-GTTCACTTTCCTGTGCGTGTTGTGTCTGGC-3′ (p.Gln913Cys aka Q913C: chosen because this de novo mutant had been previously included in a cysteine scanning study and was shown to exhibit defective plasma membrane targeting:)^26^, 5′-GTTCACTTTCCTGGAGGTGTTGTGTCTGGC-3′ (pGln913Glu aka Q913E: chosen as an example of a ‘negatively charged’ residue), 5′-GTTCACTTTCCTGAAGGTGTTGTGTCTGGC-3′ (p.Gln913Lys aka Q913K: chosen as a second example of a ‘positively charged’ residue), 5′-GTTCACTTTCCTGCTGGTGTTGTGTCTGGC-3′ (pGln913Leu aka Q913L: chosen as an example of a ‘nonpolar, aliphatic’ residue), and 5′-GTTCACTTTCCTGAGCGTGTTGTGTCTGGC-3′ (p.Gln913Ser aka Q913S: chosen as an example of a ‘polar, uncharged’ residue). Synthesis of oligonucleotide primers and sequencing of cDNA clones was performed by Eurofins Genomics (Huntsville, AL).

### Solutions

Ca^2+^-free wash-buffer contains (in mM): 82 NaCl, 2 KCl, 20 MgCl_2_, 5 HEPES, adjusted to pH 7.45 with NaOH.

ND96 ‘Na (0 HCO_3_)’ solution contains (in mM): 93.5 NaCl, 2 KCl, 1.8 MgCl_2_, 1 CaCl_2_, 5 HEPES, adjusted to pH 7.5 with fresh (i.e. nominally HCO_3_^−^-free) NaOH solution.

NaHCO_3_ ‘Na, HCO_3_’ solution: (in mM): 60.5 NaCl, 33 NaHCO_3_, 2 KCl, 1.8 MgCl_2_, 1 CaCl_2_, 5 HEPES. Equilibrated to pH 7.5 by bubbling with 5% CO_2_/21% O_2_/balance N_2_ generated from component gases using a Series 4000 gas mixing system (Environics, Tolland, CT).

Na^+^-free HCO_3_^−^ ‘(0 Na) HCO_3_’ solution: (in mM): 60.5 NMDG.Cl, 33 NMDG.HCO_3_, 2 KCl, 1.8 MgCl_2_, 1 CaCl_2_, 5 HEPES. Equilibrated to pH 7.5 by bubbling with 5% CO_2_/21% O_2_/balance N_2_. (NMDG: *N*-methyl-D-glucamine).

In Cl^−^ replacement solutions, Na-Gluconate substitutes for Cl^−^ salts and we doubled the concentration of Mg^2+^ and Ca^2+^ salts as a compensation for divalent-cation chelation. DIDS-containing solutions (4,4′-diisothiocyanato-2,2′-stilbenedisulfonate: Sigma-Aldrich) were used on the day of preparation and covered in aluminum foil to minimize exposure to light.

OR3 medium (14 g/L of powdered Leibovitz’s L-15 medium (Thermo Fisher Scientific, Rockford, IL), 100 units/mL penicillin, 100 µg/mL streptomycin, 5 mM HEPES, adjusted to pH 7.5 using NaOH.

The osmolality of all solutions and media (except for Ca^2+^-free wash buffer) was corrected to 195 ± 5 mmol/kg with H_2_O, as measured using a Vapro vapor pressure osmometer (Wescor, Logan, UT).

### Preparation and culture of Xenopus oocytes

Extraction of ovaries from *Xenopus laevis* frogs was approved by and performed in accordance with the rules and recommendations of the Institutional Animal Care and Use Committee (IACUC) at the University at Buffalo.

*Xenopus laevis* frogs were anesthetized in a 0.2% solution of pharmaceutical grade Tricaine-S (Western Chemical Inc., Ferndale, WA). Frogs were euthanized by exsanguination following surgical extraction of their ovaries. The excised ovaries were cut into ~5mm^2^ fragments and washed for 3 × 5 min in Ca^2+^-free wash-buffer. Individual oocytes were released from the ovary and defolliculated during a 30 min exposure of the washed fragments to Ca^2+^-free wash-buffer containing 2 mg/mL type-IA collagenase (Sigma-Aldrich, St Louis, MO) solution. Following two further 5-min washes in Ca^2+^-free wash buffer, and one wash in ND96 solution (see Electrophysiology sections), the oocytes were re-suspended in OR3 medium and maintained at 18 °C prior to H_2_O/cRNA injection.

### NBCe1-A expression in oocytes

pGH19 vector constructs were linearized with *Not*I at 37 °C for 2 hours and purified using a MinElute PCR Purification Kit (QIAgen, Valencia, CA) for use as template in *in vitro* transcription reactions. cRNA was synthesized using a T7 mMessage mMachine transcription kit (Life Technologies, Grand Island, NY), purified using an RNeasy MinElute cleanup kit (QIAgen), and quantified using a Nanodrop 2000 spectrophotometer (Thermo Fisher Scientific). Individual oocytes were injected with 25 nL of 1000 ng/nL cRNA or 25 nL of H_2_O and maintained at 18 °C in OR3 medium for 3–5 days prior to experimentation.

Note that all NBCe1 clones used in this study included a C-terminal EGFP tag, although the EGFP suffix is omitted from their mention in the text and figures to aid readability.

### Biotinylation and western blotting

Biotinylation experiments were performed on groups of 15 oocytes using the Pierce Cell Surface Protein Isolation Kit (Thermo Fisher Scientific) according to the manufacturer’s instructions, with the difference that solutions applied to intact cells were diluted to 195 ± 5 mmol/kg osmolality to be isosmotic with *Xenopus* plasma. Biotinylated (membrane isolated) fractions of oocytes were resolved on 3–8% Tris-Acetate protein gels (Thermo Fisher Scientific) and transferred onto PVDF membranes using an XCell II Blot Module (Thermo Fisher Scientific). Resolved NBCe1-A protein was visualized by blocking the membrane with TBS-T containing 2% milk powder for 1 hour, exposing the membrane to an anti-EGFP monoclonal antibody (‘JL-8’ at 1:5000 dilution: Clontech Laboratories, Mountain View, CA) for 1 hour, a horse-radish-peroxidase (HRP)-conjugated goat anti-mouse secondary antibody (#55563 at 1:2000 dilution: MP Biomedicals, Solon, OH) for 1 hour, and ECL2 chemiluminescent substrate (Thermo Fisher Scientific) for 5 min. Images were acquired using a Pierce MyECL imager (Thermo Fisher Scientific) and analysed using Fiji software^27^. Both bands (Fig. 5B) were included in the densitometric analysis: the lower band is monomeric NBCe1-A-EGFP, the upper band represents a pool of NBCe1-A-EGFP that retains its dimeric status during SDS-solubilization.

### Electrophysiology

Current-passing and voltage-sensing microelectrodes for voltage clamp were pulled from Clark borosilicate capillary glass (#BF200-156-10, Sutter Instruments, Novato, CA) using a P-1000 micropipette puller (Sutter Instruments). The tips of the microelectrodes were filled with saturated KCl (#SP138-500, Thermo Fisher Scientific) and the filled electrodes were mounted in Ag/AgCl_2_ half-cells that connect the liquid junction to OC-275C oocyte clamp circuitry (Warner Instruments) via a silver wire. The resistance of these electrodes was 0.5–1.0 MΩ. Individual oocytes were placed in a perfusion chamber (#RC-3Z, Warner Instruments, Hamden, CT) and impaled with both electrodes. Oocyte membranes were clamped to their spontaneous electrical potential (*V*_*m*_) prior to initiating a protocol (under the control of pClamp 10.4 software: Molecular Devices, Sunnyvale, CA) in which the potential was stepped to +120 mV for 100 ms, returned to *V*_*m*_ for 100 ms and then stepped to +100 mV and so on for 15 × 20 mV increments. Perfusion solutions were interchanged using a six channel perfusion pinch-valve control system (#VC-6, Warner Instruments). Oocytes were released from clamp prior to any solution change followed by re-clamping after 1 min after solution change, unless the solutions contained the NBCe1-blocker DIDS, in which case they were allowed 2 min before re-clamping to allow additional time for DIDS interaction. Current-voltage relationships (I-V plots) were generated using Clampfit 9.0 software (Molecular Devices).

Slope conductances were calculated from I-V plots over the range −20 mV to +20 mV range, a region over which endogenous conductances interfere least with NBCe1-mediated conductances.

### Mammalian-cell culture, transfection, and fluorescence microscopy

Madine Derby Canine Kidney cells (MDCK-II, Sigma, passage number 2–4) were used as a model for polarised renal epithelia. MDCK-II cells were cultured using antibiotic free Minimum Essential Medium with L-glutamine (#11095–072, Thermo Fisher Scientific) supplemented with 5% heat-inactivated fetal bovine serum (Denville Scientific Inc., Holliston, MA) and grown in T-75 flasks at 37 °C in 5% CO_2_/95% air. Cells were released from the flasks using 0.05% trypsin-EDTA (Thermo Fisher Scientific, Waltham, MA) digest and seeded at 1 × 10^5^ cells/well into Lab-Tek® II CC2™ 4-chambered slides (Electron Microscopy Sciences, Hatfield, PA). Once the monolayer had achieved 80–90% confluence, cells were bathed for 30 min in 2 mM EGTA at 37 °C^28^ and transiently transfected with NBCe1-A-EGFP.pcDNA3.1 constructs using Lipofectamine 3000 (Invitrogen, Carlsbad, CA) following the manufacturer’s protocol. 48 h later, expression of NBCe1-A-EGFP was confirmed by visualizing EGFP fluorescence using a ZOE Fluorescent Cell Imager (Bio-Rad Laboratories, Hercules, CA). Transfected monolayers were fixed and permeabilized by a 5 min incubation in 60% methanol/40% acetone mixture at −20 °C. Monolayers were blocked with PBS + 4% BSA for 15 min and washed with PBS for 3 × 5 min. Basolateral membranes were visualized using a mouse anti-Na^+^/K^+^-ATPase antibody (#C464.6 at 1:1000 dilution: EMD Millipore, Billerica, MA) followed by an Alexa594-conjugated goat-anti-mouse secondary antibody (#A-11032 at 1:200 dilution: Thermo Fisher Scientific). NBCe1-A-EGFP distribution was visualized using a rabbit anti-GFP antibody (#A-11122 at 1:100 dilution: Thermo Fisher Scientific) followed by an Alexa488-conjugated goat-anti-rabbit secondary antibody (#A-11034 at 1:500 dilution: Thermo Fisher Scientific). X-Y images and Z-stack sections (0.24 µM separation) were obtained using a Zeiss Axio Imager 2 fluorescence microscope with Apotome.2 attachment for optical sectioning (Carl Zeiss, Jena, Germany). Images were acquired using ZenPro software. Images were analysed using Fiji software. Pearson’s coefficients for co-localization analysis were calculated using the ‘Just Another Localization Plugin (JAcoP) for Fiji. For each cell, a 4–12 µm^2^ area of interest was selected around a section of membrane (identified by Na,K-ATPase immunofluorescence) in the X-Y plane and the co-incidence of EGFP immunofluorescence was with Na,K-ATPase immunofluorescence was reported for each pixel in that area throughout the Z-stack.

### Homology model

The model was generated using Phyre2 (protein homology/analogy recognition engine v 2.0:)^29^ at the online Phyre2 protein fold recognition server. The amino acid sequence of the transmembrane domain of human NBCe1 was input to generate homology models using the Phyre2 intensive modeling mode. The model presented here is modeled on the crystal structure of the transmembrane domain of SLC4A1 (PDB ID: 4ZYF:)^18^. The model was visualized and annotated using Swiss-Pdb Viewer 4.1.0^30^.

### Statistical Analysis

Microsoft Excel 2013 was used for t-tests. Minitab 17 (Minitab Inc., State College, PA) was used for multiple comparisons (One-Way ANOVA with Tukey’s post hoc analysis). Both analyses use a 95% confidence limit. For instances in which it was appropriate to perform multiple t-tests, Bonferroni correction was applied to the confidence limit (i.e., increasing the confidence limit by a factor that equals the number of t-tests, n: P = 0.05/n).

### Data Availability

The datasets generated during and/or analysed during the current study are available from the corresponding author on reasonable request.
